# Proximity-Based Compression for Network Embedding

**DOI:** 10.3389/fdata.2020.608043

**Published:** 2021-01-26

**Authors:** Muhammad Ifte Islam, Farhan Tanvir, Ginger Johnson, Esra Akbas, Mehmet Emin Aktas

**Affiliations:** ^1^ Department of Computer Science, Oklahoma State University, Stillwater, OK, United States; ^2^ Department of Computer Science, University of Tulsa, Tulsa, OK, United States; ^3^ Department of Mathematics and Statistics, University of Central Oklahoma, Edmond, OK, United States

**Keywords:** network embedding, graph representation learning, graph compression, graph classification, node similarity

## Abstract

Network embedding that encodes structural information of graphs into a low-dimensional vector space has been proven to be essential for network analysis applications, including node classification and community detection. Although recent methods show promising performance for various applications, graph embedding still has some challenges; either the huge size of graphs may hinder a direct application of the existing network embedding method to them, or they suffer compromises in accuracy from locality and noise. In this paper, we propose a novel **N**etwork **E**mbedding method, NECL, to generate embedding more efficiently or effectively. Our goal is to answer the following two questions: 1) Does the network **C**ompression significantly boost **L**earning? 2) Does network compression improve the quality of the representation? For these goals, first, we propose a novel graph compression method based on the neighborhood similarity that compresses the input graph to a smaller graph with incorporating local proximity of its vertices into super-nodes; second, we employ the compressed graph for network embedding instead of the original large graph to bring down the embedding cost and also to capture the global structure of the original graph; third, we refine the embeddings from the compressed graph to the original graph. NECL is a general meta-strategy that improves the efficiency and effectiveness of many state-of-the-art graph embedding algorithms based on node proximity, including DeepWalk, Node2vec, and LINE. Extensive experiments validate the efficiency and effectiveness of our method, which decreases embedding time and improves classification accuracy as evaluated on single and multi-label classification tasks with large real-world graphs.

## 1 Introduction

Networks are effectively used to represent relationships and dependence among data. Node classification, community detection, and link prediction are some of the applications of network analysis in many different areas such as social networks and biological networks. On the other hand, there are some challenges in network analysis, such as high computational complexity, low parallelizability, and inapplicability of machine learning methods (Cui et al., 2018). Recently, network embedding as representation learning from graph has become popular for many problems in network analysis (Hamilton et al., 2017; Zhang et al., 2017; Cai et al., 2018; Cui et al., 2018; Goyal and Ferrara, 2018). Network embedding is defined as encoding structural information of graphs into a low-dimensional vector space on their connections (Perozzi et al., 2014). By preserving structure information of the network, nodes with links will be close to each other in vector space. While desirable network embedding methods for real-world networks should preserve the local proximity between vertices and the global structure of the graph, it should also be scalable for large networks (Tang et al., 2015).

While early methods, which consider the network embedding as a dimensionality reduction (Belkin and Niyogi, 2001), are effective on small graphs, the major concern of them is that time complexity is at least quadratic in the number of graph vertices. Therefore, it is not possible to apply them on large-scale networks with billions of vertices (Zhang et al., 2017; Cai et al., 2018; Cui et al., 2018). In recent years, more scalable methods that use matrix factorization or neural networks have been proposed with transforming the network embedding problem into an optimization problem (Tang et al., 2015). DeepWalk (Perozzi et al., 2014) is the pioneering work that uses the idea of word representation learning (Mikolov et al., 2013a; Mikolov et al., 2013b) for network embedding. They preserve network structures or local neighborhood proximity with path sampling using short random walks (Perozzi et al., 2014; Grover and Leskovec, 2016). With path sampling, network embedding is converted to word embedding with considers random walk as a sequence of words. Therefore, it is expected that vertices in a similar neighborhood get similar paths and hence similar representations.

Although recent methods show promising performance for various applications, graph embedding still has some challenges. First of all, many of these methods are still computationally expensive and need a large amount of memory, so they are not scalable to large graphs (scalability problem). Secondly, these approaches attempt to address the non-convex optimization goal using stochastic gradient descent, hence optimization on the co-occurrence probability of the vertices can easily get stuck at a bad local minima as the result of poor initialization (initialization problem). This may cause generating dissimilar representations for vertices within the same or similar neighborhood set. Also, many of these methods use local information with short random walks during the embedding by ignoring the global structure in the graph.

These challenges have motivated researchers to use graph compression (summarization) algorithm that reduces the complexity and size of large graphs. The aim of graph compression is to create a smaller supergraph from a massive graph such that the crucial information of the original graph will be maintained in the supergraph. Vertices with similar characteristics are grouped and represented by super-nodes. Approximations with compressing are used to solve original problems more efficiently, such as all-pairs shortest paths, search engine storage, and retrieval (Adler and Mitzenmacher, 2001; Suel and Yuan, 2001). Using an approximation of the original graph not only makes a complex problem simpler but also makes a good initialization to solve the problem. It has been proved successful in various graph theory problems (Gilbert and Levchenko, 2004). For the scalability problem, embedding on the coarsest graph is more efficient and needs far less memory that makes existing embedding methods applicable to large graphs. For the initialization problem, grouping vertices with similar characteristics in a compressed graph solves the problem of getting different representations for them.

HARP (Chen et al., 2018b) addresses the initialization problem by hierarchically compressing the graph by combining nodes into super-nodes randomly. Thus, it produces effective, low-level representation of nodes though multi-level learning. However, random edge compressing may put dissimilar nodes into the same super-node making their representation similar. Also, multi-level compressing and learning result in significant compression and embedding cost, hence HARP fails to address the scalability problem.

In this paper, we use graph compression to address these two problems and also the limitations of HARP. More precisely, we study graph compression for the Network Embedding problem to answer these two questions:

Does the Network Compression Significantly Boost Learning?

Does the Network Compression Improve the Quality of the Representation?

Our main goal is to obtain more *efficient* and more *effective* network embedding models as answers to these questions. For this goal, we present an extension of our first method, NECL, that is a general meta-strategy for network embedding. We propose a proximity-based graph compression method that compresses the input graph to a smaller graph with incorporating the neighborhood similarity of its vertices into super-nodes. NECL compresses the graph by merging vertices with similar neighbors into super-nodes instead of random edge merging, as HARP does. NECL employs the embedding of the compressed graph to obtain the embedding of the original graph. This brings down the embedding cost and captures the global structure of the original graph without losing locality kept in the super-nodes. In addition to reducing the graph’s size for embedding, we get less pairwise relationships from random walks on a smaller set of super-nodes, which generates less diverse training data for the embedding part. All these facts improve efficiency while maintaining *similar* or *better effectiveness* comparing to the baseline methods. We then project the embedding of super-nodes to the original nodes.

In NECL, we primarily focus on improving the efficiency of embedding methods, so we do not employ refinement. As a result, we may lose some local information of the nodes because of merging. To overcome this problem, in this paper, we go beyond our original NECL by introducing an embedding refinement method NECL-RF. Our second method, NECL-RF, uses the compressed graph’s projected embedding to initialize the representation for the original graph embedding. Refining these initial representations aids in learning the original graph’s embedding. This provides global information of the graph into learning and also solves the different initialization problem of similar vertices, hence increases the effectiveness. Since the compressed graph is quite small compared to the original graph, the learning time will not increase significantly. Hence *similar efficiency* is maintained compared to the baseline methods. Moreover, we provide a richer set of experiments to evaluate NECL and NECL-RF. While in the earlier version, we only use DeepWalk and Node2vec as baseline methods for representation learning and combine them with NECL as a general meta-strategy. In this paper, we add one more baseline method, LINE, and present the results of all with NECL and NECL-RF.

**FIGURE 1:**
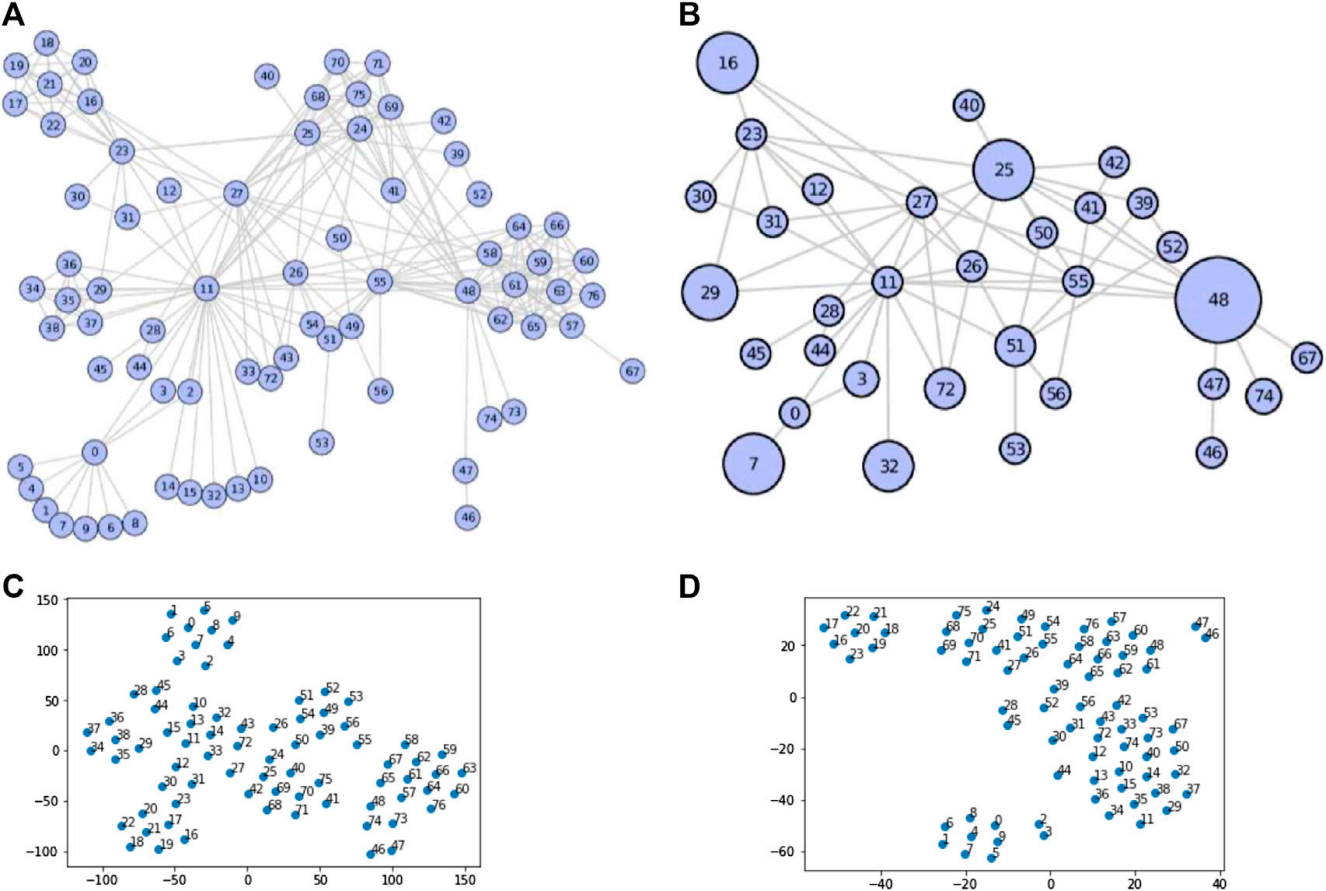
Example of graph compressing on Les Miserables network (Original Network **(A)**, Compressed Network **(B)**, Embedding of Original Network **(C)** and Embedding of Compressed Network **(D)**).

Example **1.** In Figure 1, we present the effectiveness of our compressing and embedding model,NECL, on the well-known Les Miserables network. This undirected network contains co-occurrences of characters in Victor Hugo’s novel ‘Les Miserables’. A node represents a character and an edge between two nodes shows that these two characters appeared in the same chapter of the book. While the original network has 77 vertices and 254 edges, the compressed network has 33 vertices and 64 edges. As we see in the figure, the compressed network preserves the local structure of vertices in super-nodes without losing the global structure of the graphs. It is expected that nodes close in a graph should also be close in the embedding space. For example, in Figure 1A neighborhood sets of the vertices {1,4,5,6,7,8,9} are same and including just node 0. Hence, random walks from these vertices have to pass from node 0 and get very similar walks and so very similar embedding. Instead of walking separately from each of these vertices, we just need to walk for the super-node seven in the compressed graph in Figure 1B and learn one embedding. As presented in Figures 1C,D, as the embedding of nodes with the original graph (C) and compressed graph (D), node proximity is preserved in the compressed graph. So, nodes close in original graph embedding are also close in compressed graph embedding.

We summarize our contributions as follows.

• New proximity-based graph compressing method: Based on the observation that vertices with similar neighborhood sets get similar random walks and eventually similar representation, we merge these vertices into super-nodes to get a smaller compressed graph that preserves the proximity of nodes in the original large graph.
• Efficient embedding without losing effectiveness: We do random walks and embedding on the compressed graph, which is much smaller than the original graph, efficiently. This method has similar effectiveness with baseline methods by preserving the global and local structure of the graph in the compressed graph.
• Effective embedding without decreasing efficiency: We use the embedding obtained from the compressed graph as initial vectors for the original graph embedding. This combines the global and local structure of the graph and improves the effectiveness. Embedding of a small compressed graph does not take much time with respect to original graph embedding, so it will not increase the embedding time significantly.
• Generalizable: NECL is a general meta-strategy that can be used to improve the efficiency and effectiveness of many state-of-the-art graph embedding methods. We report the results for DeepWalk Node2vec and LINE.

• The paper is structured as follows. In Section 2, we give the necessary background for our method. and also provide related work. In Section 3, we introduce our neighborhood similarity-based graph compression model by explaining our similarity measure and two different embedding methods that use the compressed graph. In Section 4, we present our experimental results and compare them with the baseline methods. Our final remarks are reported in Section 5.

## 2 Background

In this section, we discuss related works in the area of network embedding. We give some details of pioneer works in network embedding focusing on DeepWalk. We also explain random walk based sampling methods and multi-level network embedding approaches here.

### 2.1 Network Embedding

Network embedding plays a significant role in network data analysis, and it has received huge research attention in recent years. Previous researchers consider the graph embedding as a dimensionality reduction (Chen et al., 2018a), such as PCA (Wold et al., 1987) that captures linear structural information and LE (locally linear embeddings) (Roweis and Saul, 2000) that preserves the global structure of non-linear manifolds. While these methods are effective on small graphs, scalability is the major concern with them being applied to large-scale networks with billions of vertices, since the time complexity of these methods is at least quadratic in the number of graph vertices (Zhang et al., 2017; Wang et al., 2018). On the other hand, recent approaches in graph representation learning focus on the scalable methods that use matrix factorization (Qiu et al., 2018; Sun et al., 2019) or neural networks (Tang et al., 2015; Cao et al., 2016; Tsitsulin et al., 2018; Ying et al., 2018). Many of these aim to preserve the first and second-order proximity as a local neighborhood with path sampling using short random walks such as DeepWalk and Node2vec (Hamilton et al., 2017; Cai et al., 2018; Cui et al., 2018; Goyal and Ferrara, 2018). Some recent studies aim to preserve higher-order proximity (Ou et al., 2016; Chen et al., 2018b). In addition to these, some recent works integrate contents to learn better representations (Akbas and Zhao, 2019). While some studies use network embedding on node and graph classification (Perozzi et al., 2014; Niepert et al., 2016; Chen et al., 2018b), some others use it on graph clustering (Cao et al., 2015; Akbas and Zhao, 2019; Akbas and Zhao, 2017).

DeepWalk (Perozzi et al., 2014) is the pioneering work that uses the idea of word representation learning in (Mikolov et al., 2013a; Mikolov et al., 2013b) for network embedding. While vertices in a graph are considered as words, neighbors are considered as their context in natural language. A graph is represented as a set of random walk paths sampled from it. The learning process leverages the co-occurrence probability of the vertices that appear within a window in a sampled path. The Skip-gram model is trained on the random walks to learn the node representation (Mikolov et al., 2013a; Mikolov et al., 2013b). We give the formal definition of network embedding as follows.

Definition 1 (Network embedding). *Network embedding is a mapping* $\phi :V\to {\mathbb{R}}^{d},d\ll |V|$ *which represents each vertex* v∈V *as a point in a low dimensional space* ${\mathbb{R}}^{d}$ *.*

Here *d* is a parameter specifying the number of dimensions of our node representation. For every source node u∈V, we define $N_{S}\left(u\right)\subset V$ as a network neighborhood of node *u* generated through a neighborhood sampling strategy *S*. We seek to optimize the following objective function, which maximizes the log-probability of observing a network neighborhood $N_{S}\left(u\right)$ for a node *u* conditioned on its representation, given by ϕ

$$\underset{f}{\text{max}}\underset{u\in V}{\sum }logPr\left(N_{S}\left(u\right)|\phi \left(u\right)\right).$$ (1)

There is an assumption that the conditional independence of vertices will ignore the vertex ordering in the neighborhood sampling to make the optimization problem tractable. Therefore, the likelihood is factorized by assuming that the likelihood of observing a neighborhood node is independent of observing any other neighborhood node given the representation of the source

$$Pr\left(N_{S}\left(u\right)|\phi \left(u\right)\right)=\underset{n_{i}\in N_{S}\left(u\right)}{\prod }Pr\left(n_{i}|\phi \left(u\right)\right).$$

The conditional likelihood of every source-neighborhood node pair is modeled as a softmax unit parametrized by a dot product of their features.

$$Pr\left(n_{i}|\phi \left(u\right)\right)=\frac{\text{exp}\left(\phi \left(n_{i}\right)\cdot \phi \left(u\right)\right)}{\sum_{v\in V}\text{exp}\left(\phi \left(v\right)\cdot \phi \left(u\right)\right)}.$$

It is too expensive to compute the summation over all vertices for large networks, so we approximate it using negative sampling (Mikolov et al., 2013b). We optimize Equation 1 using stochastic gradient ascent over the model parameters defining the embedding ϕ.

#### 2.1.1 Random Walk Based Sampling

The neighborhoods $N_{S}\left(u\right)$ are not restricted to just immediate neighbors but can have vastly different structures depending on the sampling strategy *S*. There are many possible neighborhood sampling strategies for vertices as a form of local search. Different neighborhoods coming from different strategies result in different learned feature representations. For scalability of learning, random walk based methods are used to capture the structural relationships of vertices. They maximize the co-occurrence probability of subsequent vertices within a fixed-length window of random walks to preserve higher-order proximity between vertices. With random walks, networks are represented as a collection of vertex sequence. In this section, we take a deeper look at the network neighborhood sampling strategy based on random walks and the proximity captured by random walks.

The co-occurrence probability of node pairs depends on the transition probabilities of vertices. Considering a graph *G*, we define adjacency matrix *A* that is symmetric for undirected graphs. For an unweighted graph, we have $A_{ij}=1$ if and only if there exists an edge from $v_{i}$ to $v_{j}$, and $A_{ij}=0$ otherwise. For a graph with adjacency matrix *A*, we can define the diagonal matrix, known as degree matrix, as $D_{ij}=\underset{k}{\sum }A_{ik}$ if i=j, and $D_{ij}=0$ otherwise. In a random walk, transition probability from one node to another depends on the degree of the vertices. The probability of leaving a node from one of its edges is split uniformly among the edges. We define this one step transition probability as *T*: $T=D^{-1}A$ where $T_{ij}$ is the probability of a transition from vertex $v_{i}$ to vertex $v_{j}$ within one step.

#### 2.1.2 Multi-Level Network Embedding

Optimization of a non-convex function in these methods could easily get stuck at a bad local minima as the result of poor initialization. Moreover, while preserving local proximities of vertices in a network, they may not preserve the global structure of the network. As a solution to these issues, a multi-level graph representation learning paradigm has been proposed (Chen et al., 2018b; Ayan Kumar Bhowmick and Meneni, 2020; Liang et al., 2018; Chenhui Deng and Zhao, 2020). HARP, is proposed in (Chen et al., 2018b) as a graph preprocessing step to get better initialization vectors. In this approach, related vertices in the network are hierarchically combined into super-nodes at varying levels of coarseness. After learning the embedding of the coarsened network with a state-of-the-art graph embedding method, the learned embedding is used as an initial value for the next level. The initialization with the embedding of the coarsened network improves the performance of the state-of-the-art methods. One of the limitations of this method is that multi-level compressing and learning results in significant compression and embedding cost. Random edge compressing may put dissimilar nodes into the same super-node that makes their representation similar.

As a more efficient solution, MILE (Liang et al., 2018) performs multi-level network embedding on large graphs using graph coarsening and refining techniques. It compresses the graph repeatedly based on Structural Equivalence Matching (SEM) and Normalized Heavy Edge Matching (NHEM). After learning the embedding of the compressed graph, they refine it efficiently through a novel graph convolution neural network to get the embedding of the original graph. This way, it receives embedding for large scale graphs in an efficient and effective way. More recently, GraphZoom (Chenhui Deng and Zhao, 2020) proposes a multi-level spectral approach to enhange both the quality and scalability. It performs graph fusion to generate a new graph that effectively encodes the topology of the original graph and the node attribute information. Then they apply spectral clustering methods to merge the nodes into super-nodes with the aim of retaining the first few eigenvectors of the graph Laplacian matrix. Finally, after getting the embedding of the compressed graph, they refine it by applying projection on it to get the original graph embedding. LouvainNE (Ayan Kumar Bhowmick and Meneni, 2020) applies the Louvain clustering algorithm recursively to partition the original graph into multiple subgraphs and construct a Hierarchy partition of the graph, which is represented as a tree. Then they generate different meta-graph from the tree and apply the baseline method i.e., DeepWalk Node2vec. After getting the embedding from different meta-graph, they combine these embeddings to find the final embedding. They use a parameter to regulate the weights of different embedding for combining.

Our approach differs from these by applying similarity-based compressing to preserve the local information. Also, all of these approaches apply hierarchical compressing that may take more time, but we apply single level compressing and use it to get the original graph embedding. NECL uses the graph coarsening to capture the local structure of the network without a hierarchical manner to improve the efficiency of the random walk based state-of-the-art methods.

## 3 Methodology

While a desirable network embedding method for real-world networks should preserve the local proximity between vertices and the global structure of the graph, it should also be scalable for large networks. This section presents our novel network embedding models, NECL and NECL-RF, which satisfy these requirements. We extend the idea of the graph compressing layout to network representation learning methods. After giving some preliminary information, we explain our proximity-based compression method and how we combine compression with network embedding.

In this paper, we consider an undirected, connected, simple graph $G=\left(V_{G};E_{G}\right)$ where $V_{G}$ is the set of vertices, and $E_{G}\subseteq \left\{V_{G}\times V_{G}\right\}$ is the set of edges. The set of neighbors for a given vertex $v\in V_{G}$ is denoted as $N_{G}\left(v\right)$, where $N_{G}\left(v\right)=\left\{u|u\in V_{G}:\left(u,v\right)\in E_{G}\right\}$. We now define what a compressed graph is.

Definition 2 (Compressed graph). *A compressed graph of a given graph* $G=\left(V_{G};E_{G}\right)$ *is represented as* CG=(S;M) *where* $S=\left(V_{S};E_{S}\right)$ *is the graph summary with super-nodes* $V_{S}$ *and super-edges* $E_{S}$ *and M is a mapping from each node v in $V_G$ to its super-node in* $V_{S}$ *. A super-edge* $E=\left(V_{i};V_{j}\right)$ *in* $E_{S}$ *represents the set of all edges between vertices in the super-nodes* $V_{i}$ *and* $V_{j}$ *.*

### 3.1 Neighborhood Similarity-Based Graph Compression

The critical problem for graph compressing with preserving local structures of the graph is to identify vertices that have similar neighborhoods accurately, so they are more likely to have similar representation. In this section, we discuss how to select vertices to merge into super-nodes.

#### 3.1.1 Motivation

The motivation of our method is that if two vertices have many common neighbors, many embedding algorithms that preserve local neighborhood information will give similar representations to them. This comes from our following observation that if two vertices, $v_{i}$ and $v_{j}$, of a graph have many common neighbors, they also have similar transition probabilities to other vertices. This means that if $A_{i}$ and $A_{j}$ are similar, their transition probability vectors, $T_{i}=A_{i}\text{*}D_{ii}^{1}$ and $T_{j}=A_{j}\text{*}D_{jj}^{1}$, will be similar as well. Hence they have similar neighborhoods and get similar neighborhood sets from random walks, and as a result, they get similar representations from the learning process.

**FIGURE 2:**
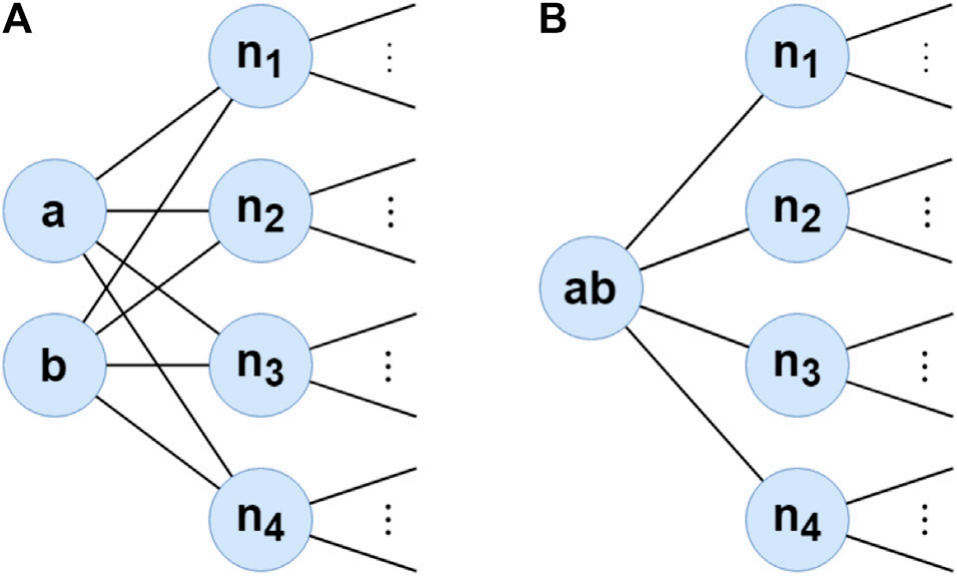
Example of graph compressing. **(a,b)** are merged into super-node ab connected to the neighbors of both **(a,b)**.

For example, in the toy graph in Figure 2, the neighbor sets of the nodes *a* and *b* are the same. Hence, their transition probabilities to the other neighbor vertices are also the same, i.e., $p\left(n_{i}|a\right)=p\left(n_{i}|b\right)=1/4$ for all i∈{1,2,3,4}. Starting on either *a* or *b* yields the same or very similar walks, so they have the same or similar representation. Therefore, instead of walking and learning representations for both *a* and *b*, it is enough to learn one for both of them. For this, we can merge this node pair (a,b) into one super-node ab. Transition probabilities of this super-node to neighbors of *a* and *b* are still the same with *a* and *b*, i.e., $p\left(n_{i}|ab\right)=1/4$ for all i∈{1,2,3,4}. When we obtain the representation of the super-node ab, we can project it as the representation of each node in this pair. Merging these vertices keeps the preservation of the first and second-order proximity. Thus, this does not affect the results of walking and learning, but it increases efficiency.

Furthermore, compressing may change the transition probability of neighbors of compressed vertices since the number of their neighbor decrease. As a result, the transition probability of each neighbor changes. For example, in the toy graph in Figure 2A, while the transition probability from $n_{1}$ to its neighbors is $\frac{1}{\left|N\left(n_{1}\right)\right|}$, after compressing, it becomes $\frac{1}{|N\left(n_{1}\right)|-1}$ since the number of neighbors decrease by one. In order to avoid this problem, we assign weights to edges of super-nodes based on the number of merged edges within the compression. For example, the super-edge between super-node ab and $n_{1}$ includes two edges which are $\left(a,n_{1}\right)$ and $\left(b,n_{1}\right)$. Therefore, the weight of the super-edge ($ab,n_{1}$) should be 2.

In a real-world graph, it is not expected to have too many vertices sharing exactly same neighborhood. However, for many graph mining problems, such as node classification and graph clustering, if two vertices share many common neighbors, they are expected to be in the same class or cluster, although their neighbor sets are not completely the same. Hence, we expect to have similar feature vectors for the vertices in the same class/cluster after embedding. From these observations, we can also apply the same merge operation on these vertices. Following the same idea in the example above, if neighbors of two vertices are similar (but not exactly the same), we can merge them into a super-node and learn one representation for all. While we can project this super-node embedding to original vertices and use the same representation for both, we can also update them in the refinement phase to embed the difference of them into their representation.

#### 3.1.2 Proximity Based Graph Compressing

In this section, we define our graph compressing algorithm formally.

For a given graph *G*, if a set of vertices $n_{1},n_{2},\ldots ,n_{r}$ in $V_{G}$ have similar neighbors, we merge these vertices into one super-node $n_{12...r}$ to get a smaller compressed graph $G^{\prime }\left(V_{G\prime },E_{G\prime }\right)$. To decide which vertices to merge, we define the *neighborhood similarity* based on the transition probability. Before defining the neighborhood similarity, we first show that cosine similarity between transition probabilities of two vertices *u* and *v*, $T_{u}$ and $T_{v}$, are determined by the number of their common neighbors.

Theorem 1. Let T be the 1-step transition probability matrix of vertices V in a graph G and let u,v∈V. Let N(u) and N(v) be the neighborhood sets of *u* and *v* and $T_{u}$ and $T_{v}$ be the transition probability vectors from u and v to other vertices. Then the similarity between $T_{u}$ and $T_{v}$ is proportional to the number of common neighbors, |N(u)∩N(v)|.

Proof. The cosine similarity between $T_{u}$ and $T_{v}$ is defined by

$$sim\left(T_{u},T_{v}\right)=\frac{\sum_{i}T_{ui}T_{vi}}{\left||T_{u}||||T_{v}|\right|}$$ (2)

By definition of *T*, we have $T_{u}=\frac{A_{u}}{\left|N\left(u\right)\right|}$ and $T_{v}=\frac{A_{v}}{\left|N\left(v\right)\right|}$. Furthermore, we have

$$||T_{u}||=1/\sqrt{\left|N\left(u\right)\right|},\;||T_{v}||=1/\sqrt{\left|N\left(v\right)\right|}$$

and

$$\sum_{i}A_{ui}A_{vi}=|N\left(u\right)\cap N\left(v\right)|.$$

Hence, if we plug in these into Equation 1, we get

$$\begin{array}{l} sim\left(T_{u},T_{v}\right)=\frac{\sum_{i}T_{ui}T_{vi}}{\left||T_{u}||||T_{v}|\right|}=\frac{\sum_{i}\frac{A_{ui}}{\left|N\left(u\right)\right|}\frac{A_{vi}}{\left|N\left(v\right)\right|}}{\frac{1}{\sqrt{\left|N\left(u\right)\right|}}\frac{1}{\sqrt{\left|N\left(v\right)\right|}}}\\ =\frac{\frac{1}{\left|N\left(u\right)||N\left(v\right)\right|}\left|N\left(u\right)\cap N\left(v\right)\right|}{\frac{1}{\sqrt{\left|N\left(u\right)||N\left(v\right)\right|}}}\\ =\frac{\left|N\left(u\right)\cap N\left(v\right)\right|}{\sqrt{\left|N\left(u\right)||N\left(v\right)\right|}}. \end{array}$$

Therefore,

$$sim\left(T_{u},T_{v}\right)\propto |N\left(u\right)\cap N\left(v\right)|$$

This finalizes the proof.

From Theorem 1, we see that the similarity of transition probabilities from two vertices to other vertices depends on the similarity of their neighbors. Therefore, for the compressing, we define the neighborhood similarity between two vertices as follows.

Definition 3 (Neighborhood similarity) Given a graph G, the neighborhood similarity between two vertices u,v is given by

$$Nsim\left(u,v\right)=\frac{2|N\left(u\right)\cap N\left(v\right)|}{\left|N\left(u\right)|+|N\left(v\right)\right|}$$ (3)

In order to normalize the effect of high degree vertices, we divide the number of common neighbors by degree of vertices. The neighborhood similarity is between 0 and 1, where it is 0 when two vertices have no common neighbor and one when both have the same neighbors. According to the neighbor similarity, we merge vertices whose similarity value is higher than a given threshold value.

The neighborhood similarity-based graph compressing algorithm is given in Algorithm 1. It is clear that the vertices with a nonzero neighborhood similarity are 2-step neighbors. Therefore, we do not need to compute the similarity between all pairs of vertices. Instead, we just need to compute the similarity between vertices and their neighbors’ neighbors. For each node $v\in V_{G}$, we compute the similarity between *v* and each *k* as neighbors of neighbors (line 3–10). Then, we check the similarity value of all pairs (*u*, *k*) in the list and if it is higher than the given threshold λ (line 12), we merge *u* and *k* into a super-node $s_{u,k}$ (line 13). Then we delete edges of *u* and *k* and add edges between neighbors of *u* and *k* and the new super-node $s_{u,k}$ (line 17–24). We assign weights to the edges of super-nodes based on the number of merged edges within the compression. Threshold λ decides the trade-off between efficiency and effectiveness. If we use a larger value, it will merge a less number of vertices. On the other hand, if we use a smaller value, we merge more vertices, and as a side effect, we may merge some dissimilar vertices as well, which may result in an increase in efficiency but cause a decrease in accuracy. Note that since we use original neighborhood similarity, the order of merging does not affect the result, so we randomly select a node and check neighbors for compression. Furthermore, one super-node may include more than two vertices of the original graph. For example, if the similarity between the vertices *x* and *y*, NSim(x,y), and the vertices *y* and *z*, NSim(y,z), are both bigger than given threshold, we merge *x* and *y* in $s_{x,y}$ and then we merge $s_{x,y}$ and *z* into $s_{x,y,z}$. Therefore, during the merge operation, we check whether the node *y* is merged with another node and if so, we get the super-node of the original node *x*.

**Algorithm 1:**
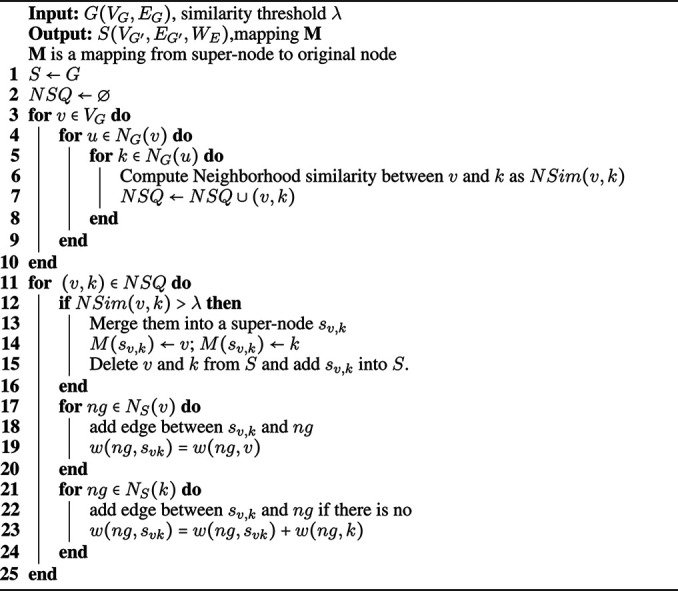
Graph Compressing (*G*, λ).

### 3.2 Network Embedding

Our NECL framework is adaptive with any embedding method which preserves the neighborhood proximity of nodes, i.e., DeepWalk, Node2vec, and LINE. We get the embedding for the original graph in two ways.

#### 3.2.1 Network Embedding on Compressed Graph

Our main goal in this section is to *improve the efficiency* of the embedding problem while maintaining *similar effectiveness* with the baseline methods. For this goal, instead of embedding the original graph, we embed the compressed graph and employ this embedding for the original graph embedding.

We first start compressing the graph for a given similarity threshold, as explained in the previous section. Then we learn the embedding of super-nodes in the compressed graph. Next, we assign the representation of each super-node in the compressed graph as the representation of the corresponding vertices in each super-node and obtain the embedding of the original graph. Since the size of the compressed graph is much smaller than the original graph, the embedding will be more efficient. The details of our algorithm for network embedding on a compressed graph is given in Algorithm 2.

**Algorithm 2:**
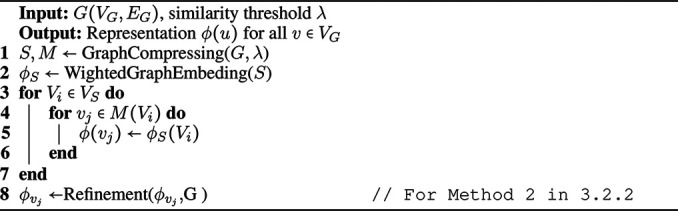
NECL: Network Embedding on Compressed Graph.

In the algorithm, after getting the weighted compressed graph *S* (line 1), we obtain the representation of super-nodes $V_{S}$ as $\phi_{s}$ in the compressed graph with the provided network embedding algorithm (line 2). We apply any random walk based representation learning algorithm on the compressed graph. We just need to apply weighted random walks to consider the edge weights. As the size of the compressed graph is smaller than the original graph, it is more efficient to get embeddings of super-nodes than single vertices. Finally, we assign the embedding of super-nodes to vertices according to the mapping *M* obtained from the compression (line 3–7). While we may lose some local information with assigning the same representation to multiple vertices, we gain efficiency. Also, we may not need to get small differences between nodes for many applications, e.g., classification, as we see in Section 4.

#### 3.2.2 Network Embedding With Refinement

Our main goal in this section is to *improve the effectiveness* of the embedding problem while still maintaining *similar efficiency* with the baseline methods. For this goal, we employ the embedding of the compressed graph as initialization to the original graph embedding and refine it.

When we compress a graph using the neighborhood similarity score, we can easily capture the global structure of the original graph. On a large original graph, the random walk may get stuck in a local neighborhood. As a result, the embedding method may not capture the global structure of the original graph. However, when we do the random walk on the compressed graph, it visits the globally similar neighbors nodes. Hence, we can capture the global proximity of the nodes. That is why, in this method, we first embed the compressed graph for a given similarity threshold to encode the original graph’s global structure in the representation as in Section 3.2.1. Then, for the embedding of the original graph, instead of starting with randomly initialized representations, which happens in the original embedding methods such as DeepWalk and Node2vec, we start with the representations obtained from the compressed graph. In the case of random representations, for example, two similar nodes are likely to have two very different and distanced representations, hence the optimization process may not provide an accurate representation and this may decrease the quality or it may take a longer time to make them similar. However, initializing the representation using the compressed graph embedding provides global structure information as an initial knowledge to the embedding. The original graph embedding updates this initial embedding with local information that may be lost with compressing. Therefore, final embeddings have better quality with integrating local and global information in one representation. In Algorithm 2, the original graph embedding is obtained in line eight by refining the compressed graph embedding given as the initial representation.

## 4 Experiments

We do our experimental studies to compare our methods with different models in terms of efficiency and effectiveness. We evaluate the quality of embeddings through challenging multi-class and multi-label classification tasks on four popular real-world graph datasets. First, in Section 4.1, we present our model’s performance based on different parameters. Then, we compare the results of our models with the results of HARP.

**Datasets:** We consider four real-world graphs^1^, which have been widely adopted in the network embedding studies. Two of them are single-label, which are Wiki and Citeseer, and two of them are multi-label datasets, which are DBLP and BlogCatalog (BlogC). In single-label datasets, each node in the datasets has a single-label from multi-class values. In multi-label datasets, a node can belong to more than one class.

**Baseline methods:** To demonstrate that our methods can work with different graph embedding methods, we use three popular graph embedding methods, namely DeepWalk, Node2vec and LINE, as the baseline methods in our model. We combine each baseline method with our methods and compare their performance. We give a brief explanation of the baseline methods in Section 2. We named our first method as NECL, which uses a compressed graph embedding as the original graph embedding, and the second method as NECL-RF, which uses the compressed graph embedding as the initial vector for original graph embedding and refine it with the original graph.

**Parameter Settings:** For DeepWalk, Node2vec, NECL(DW), NECL(N2V), NECL-RF(DW) and NECL-RF(N2V), we set the following parameters: the number of random walks γ, walk length *t*, window size *w* for the Skip-gram model and representation size *d*. The parameter setting for all models is γ=40, t=10, w=10, d=128. The initial learning rate and final learning rate are set to 0.025 and 0.001 respectively in all models. Representation size for LINE is d=64 for all model.

**Classification** We present our results and compare them with the baseline methods and also HARP in single-label and multi-label classification tasks. For the single classification task, the multi-class SVM is employed as the classifier, which uses the one-vs-rest scheme. For the multi-label classification task, we train a one-vs-rest logistic regression model with $L_{2}$ regularization on the graph embeddings for prediction. The logistic regression model is implemented with LibLinear (Fan et al., 2008).

For the evaluation, after getting embeddings for nodes in the graph, we use these embeddings as the features of the nodes. Then, we train a classifier using these features. To train the classifier, we randomly sample a certain portion of labeled vertices from the graph and use the rest of the vertices as the test data. To have a detailed comparison of methods, we vary our training ratio from 1% to 50% on the Citeseer, Wiki, and DBLP datasets and from 10% to 80% for BlogCatalog. We use larger portion training data for the BlogCatalog dataset because the number of class labels of BlogCatalog is about ten times other graphs.

We repeat the classification tasks ten times to ensure the reliability of our experiment and report the average macro $F_{1}$ and micro $F_{1}$ scores and embedding times of our models with different parameter. Since our focus is improving the efficiency of embeddings, we report the time for embedding and do not include compression time. However, as we explain in the methodology section, we just need to compute the similarity between vertices and their neighbors’ neighbors and combine them into supernodes. Furthermore, the computation is not multi-level, just one-time computation. Therefore, the compression part does not have high complexity and it does not have an impact on efficiency. All experiments are performed on a server running Ubuntu 14:04 with four Intel 2.6 GHz ten-core CPUs and 48 GB of memory. All data and code are publicly available through this link: https://github.com/esraabil/NECL.

### 4.1 Analysis of NECL

**FIGURE 3:**
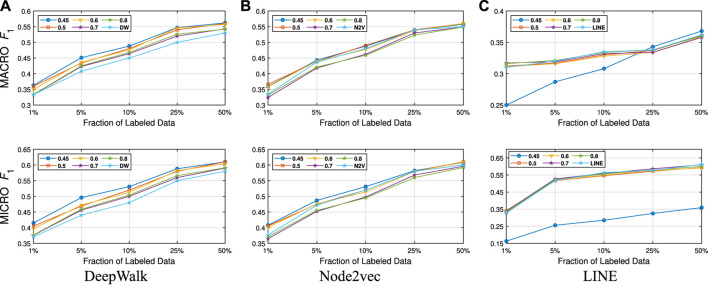
Detailed classification results on Citeseer.

**FIGURE 4:**
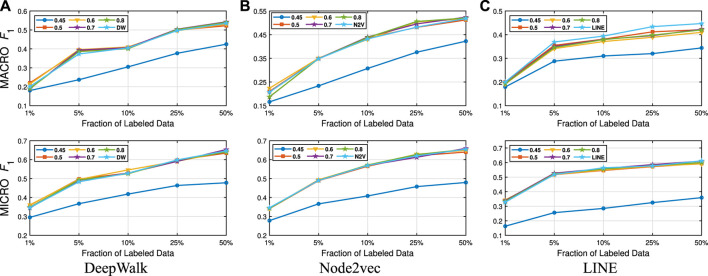
Detailed classification results on Wiki.

**FIGURE 5:**
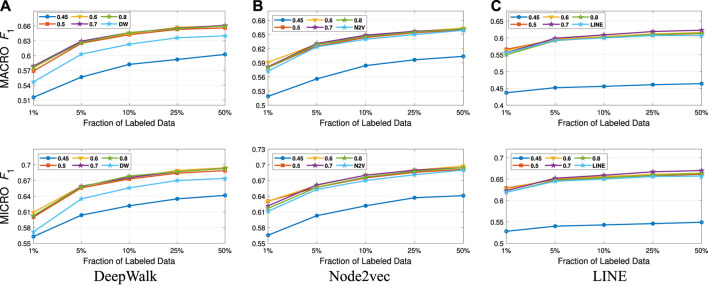
Detailed classification results on DBLP.

**FIGURE 6:**
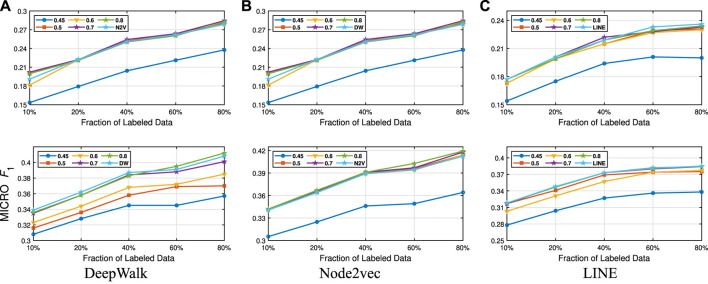
Detailed classification results on BlogCatalog.

**FIGURE 7:**
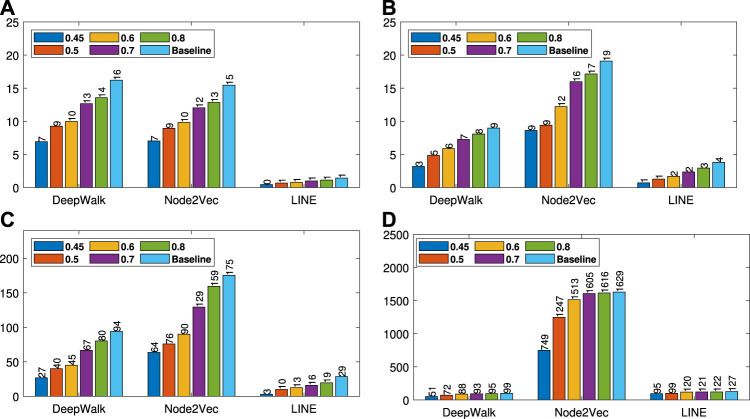
Run time analyses for different similarity threshold values λ (Citeseer **(A)**, Wiki **(B)**, DBLP **(C)** and BlogCatalog **(D)**).

**FIGURE 8:**
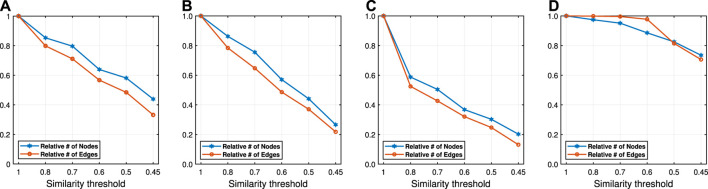
The ratio of vertices/edges of the compressed graphs to that of the original graphs. (Citeseer **(A)**, Wiki **(B)**, DBLP **(C)** and BlogCatalog **(D)**).

We present our results in Tables 1, 2. For the similarity threshold λ<0.5, the compressed graph becomes very small and gives low macro $F_{1}$ and micro $F_{1}$ scores. Since it also merges more nodes into super-nodes with a low similarity value, this may result in information loss on the graph. Hence, we set the cutting point of compression at λ=0.5. Moreover, to see the effect of the similarity threshold value λ on the compression and accuracy, we vary it from 0.45 to 1. We present the macro $F_{1}$ and micro $F_{1}$ scores with respect to the fraction of labeled data in Figures 3–6 and embedding times in Figure 7. We also report the number of edges and vertices in the compressed graph with respect to similarity threshold λ on Figure 8 to see the effectiveness of the graph compression algorithm.

**TABLE 1:** Performance comparisons of NECL with baseline methods (BL).

		Macro F_1_			Micro F_1_			Time (s)		
		NECL	BL	Gain%	NECL	BL	Gain%	NECL	BL	Gain%
	DW	0.434	0.408	6.4	0.469	0.440	6.6	9.26	16.21	42.9
	N2V	0.439	0.437	0.5	0.475	0.472	0.6	8.95	15.46	42.1
Citeseer	Line	0.317	0.320	-0.9	0.355	0.359	-1.1	0.67	1.43	53.1
	DW	0.390	0.373	4.6	0.497	0.483	2.9	4.84	8.98	46.0
	N2V	0.349	0.348	1.0	0.489	0.490	-0.2	9.41	19.10	50.7
Wiki	Line	0.355	0.369	-3.8	0.517	0.518	0.2	1.28	3.81	66.4
	DW	0.625	0.603	3.6	0.656	0.635	3.3	39.97	93.96	57.5
	N2V	0.626	0.624	0.3	0.657	0.653	0.6	75.81	175.31	56.8
DBLP	Line	0.595	0.593	0.3	0.649	0.645	0.6	9.94	28.58	65.2
	DW	0.246	0.245	0.4	0.388	0.387	0.2	71.7	99.3	27.7
	N2V	0.252	0.251	0.3	0.391	0.389	-0.5	1,247	1,628	23.4
BlogC	Line	0.215	0.219	-1.8	0.369	0.373	-1.1	99.35	126.65	21.6

**TABLE 2:** Compression ratio with the similarity threshold λ=0.5.

	|V|			|E|		
	Compressed	Original	Ratio %	Compressed	Original	Ratio %
Citeseer	1,427	2,708	47.3	5,236	10,858	51.8
Wiki	1,060	2,405	55.9	8,584	23,192	63
DBLP	8,824	27,199	69.9	32,984	133664	75.3
BlogC	8,507	10,312	17.5	543872	667966	18.6

**Gain on baseline methods:** For all datasets, we present macro $F_{1}$ and micro $F_{1}$ scores for single and multi-label classification tasks and embedding time in Table 1 and compression ratio for edge and vertices in Table 2. We use 5% training ratio of labeled vertices for Citeseer, Wiki, and DBLP and 40% training ratio for BlogCatalog. As we see from Table 1, for DeepWalk, there is a significant gain on macro and micro $F_{1}$ in addition to gain on efficiency on Citeseer, Wiki, and DBLP. For Node2vec and LINE, while there is a significant gain on total embedding time as efficiency, there is no (significant) difference between NECL and baseline methods on macro $F_{1}$ and micro $F_{1}$ For LINE, we have a higher gain on time for all datasets.

For DBLP, gains of embedding time are much higher than other datasets. On the other hand, for BlogCatalog, gains of embedding times are less with respect to other datasets. As we see from the Tables 1, 2, the gain of embedding time depends on the compression ratio of the number of edges and vertices. With compression, the number of vertices and edges for DBLP decrease from 27,199 to 8,824 (70%) and from 13,3664 to 32,984 (75%), respectively. Therefore, embedding becomes more efficient with better or same accuracy. For BlogCatalog, the compression ratio is lower than the others, around 18%; therefore, the time gain is also lower. The reason for this is that, in DBLP, vertices have many common neighbors, so the neighborhood similarity is higher and this results in more compression. On the other hand, in BlogCatalog, vertices have less common neighbors and so a lower similarity, and this results in less compression. We can conclude that while the gain in the effectiveness of our method depends on the baseline method, the gain in efficiency of our method depends on the characteristics of the dataset.

**Detailed Analyses:** We compare the performance of NECL framework for different similarity threshold values λ that results in different compression ratios with the performance of the baseline methods. Macro $F_{1}$ and micro $F_{1}$ scores on different datasets are given on Figures 3–6 for Citeseer, Wiki, DBLP and BlogCatalog datasets, respectively. We observe that for λ>0.45, macro $F_{1}$, and micro $F_{1}$ scores for NECL are similar with or higher than baseline methods across all datasets except Citeseer. For λ<=0.45, the quality of embedding decreases dramatically and so does the accuracy of classification. The results for Citeseer depend on the baseline methods. While λ=0.45 gives better accuracy for DeepWalk and Node2vec, it gives worse for LINE.

In addition to the macro $F_{1}$ and micro $F_{1}$ scores, we also report the embedding time and compression ratio for different similarity threshold values λ in Figures 7, 8. From the figures, we see that NECL takes significantly less time compared to the baseline method. As expected, for a lower threshold value λ, the compression ratio increases, and we get a smaller compressed graph and so the embedding time decreases. As BlogCatalog has a lower compassion ratio, the embedding time is less for all three baseline methods. We observe that there is a linear relation between λ and the number of vertices and edges until λ=0.5. After this point, graph sizes change dramatically for smaller λ for Citeseer, Wiki, and DBLP, but the decrease is slow for BlogCatalog until λ=0.7. One of the reasons for this situation in BlogCatalog is that the sizes of the neighbor sets for some vertices are very large, and it is not easy to get higher similarity for a larger set. For example, for two vertices with 15 edges, 10 common neighbors can be considered to have a higher similarity. On the other hand, two vertices with 150 edges should have 100 common neighbors to get the same similarity value, which is not very common.

From these detailed analyses, we observe that smaller λ results in smaller compressed graph. As a result, embedding becomes more efficient. However, for λ<=0.45, we start to lose critical information about the graph, hence, while efficiency increases, effectiveness decreases dramatically. As a solution to this problem, we refine our results with our second method, NECL-RF.

### 4.2 Comparisons of all Methods

**FIGURE 9:**
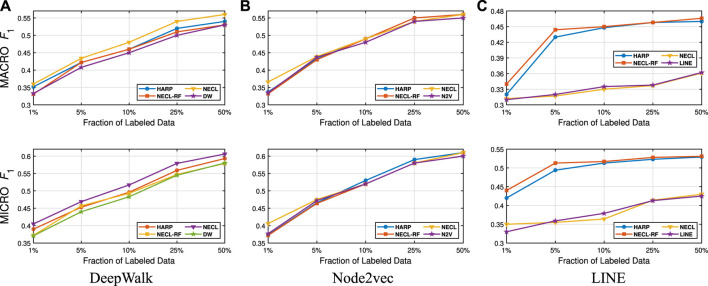
Detailed comparisons of classification results on Citeseer

**FIGURE 10:**
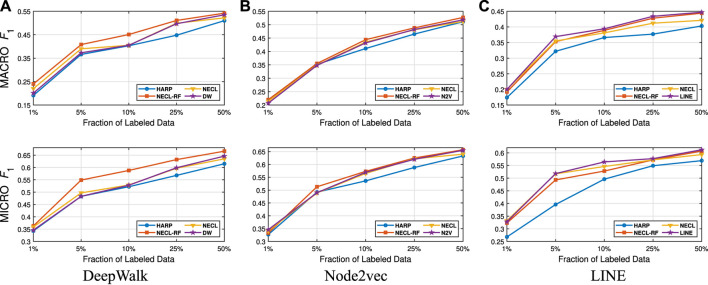
Detailed comparisons of classification results on Wiki.

**FIGURE 11:**
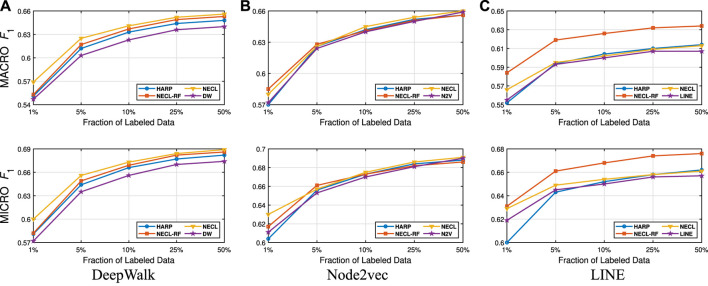
Detailed comparisons of classification results on DBLP.

**FIGURE 12:**
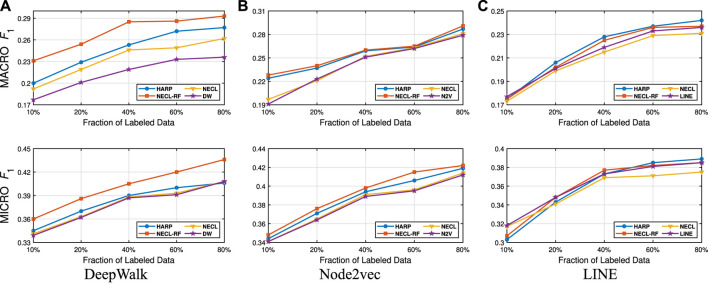
Detailed comparisons of classification results on BlogCatalog.

In this section, we evaluate the effectiveness of our NECL-RF method and compare the results with NECL, HARP, and baseline methods. From the analysis of NECL, we can see that λ=0.5 similarity threshold value gives the best result in terms of efficiency and effectiveness. For this reason, we decide to use the compressed graph for λ=0.5 threshold value and get the embedding for the compressed graph. We present the macro $F_{1}$ and micro $F_{1}$ scores achieved on all datasets in Tables 1, 2. We use 5% of the labeled vertices for Citeseer, Wiki, and DBLP, 40% for BlogCatalog as training data. To have a detailed comparison between our models, NECL and NECL-RF, HARP and the baseline methods, we vary the fraction of labeled data for classification, and present macro $F_{1}$ and micro $F_{1}$ scores in Figures 9–12.

In Table 3, we see that NECL or NECL-RF gives the highest macro $F_{1}$ and micro $F_{1}$ scores for datasets with all baseline methods except for LINE on Wiki. For DBLP, NECL or NECL-RF gives the highest accuracy for all the three baseline models. NECL-RF significantly improves the quality of the embedding for all datasets except Citeseer with Node2vec and Wiki with LINE.

**TABLE 3:** Performance comparisons of all methods.

	Citeseer		Wiki		DBLP		BlogCatalog	
	Macro F_1_	Micro F_1_	Macro F_1_	Micro F_1_	Macro F_1_	Micro F_1_	Macro F_1_	Micro F_1_
Baseline (DW)	0.408	0.440	0.373	0.483	0.603	0.635	0.245	0.387
HARP (DW)	0.422	0.453	0.366	0.483	0.612	0.644	0.253	0.390
NECL (DW)	**0.434**	**0.469**	0.390	0.497	**0.625**	**0.656**	0.246	0.388
NECL-RF (DW)	0.422	0.457	**0.408**	**0.549**	0.617	0.649	**0.285**	**0.405**
Baseline (N2V)	0.437	0.472	0.348	0.490	0.624	0.653	0.251	0.389
HARP (N2V)	0.432	0.466	0.352	0.492	0.626	0.656	0.259	0.394
NECL (N2V)	**0.439**	**0.475**	0.349	0.489	0.626	0.657	0.252	0.391
NECL-RF (N2V)	0.430	0.464	**0.372**	**0.513**	**0.628**	**0.661**	**0.260**	**0.398**
Baseline (LINE)	0.320	0.359	**0.369**	**0.518**	0.593	0.645	0.219	0.373
HARP (LINE)	0.430	0.494	0.322	0.396	0.594	0.643	0.228	0.373
NECL (LINE)	0.317	0.355	0.355	0.517	0.595	0.649	0.215	0.369
NECL-RF (LINE)	**0.444**	**0.513**	0.353	0.493	**0.619**	**0.661**	**0.252**	**0.377**

While HARP has higher accuracy than baseline methods, it does multiple levels of iteration of graph coarsening and representation learning, so it increases the time complexity. On the other hand, we do iteration only one level in NECL-RF. Embedding time for NECL-RF is the total of embedding time for the original graph and compressed graph. As we see in the previous section, the compressed graph is much smaller than an original graph, so the learning time for the compressed graph is significantly less compare to the baseline method. Hence, complexity does not increase significantly as in HARP. As a result, we get similar or better effectiveness than HARP with less time complexity.

Detailed comparisons between all methods using different portions of labeled vertices as training data are presented in Figures 9–12. In most cases, we see that in most of the cases, NECL and NECL-RF give the highest accuracy compared to other models or give better results than the baseline models. We observe that, for some datasets, refinement decreases the accuracy of NECL. The reason for this decrease might be that, for some classification tasks, learning a global structure with compressed data, which also includes a local structure in the super-nodes, would be enough. So when we relearn and update the embedding of the compressed graph, it might add noise to the features. As a result, it deteriorates the accuracy of the classification task. Also, as we see from the figures, our method has a better improvement on DeepWalk. The reason is that while Node2vec and LINE may learn higher-order proximity, regular random walk in DeepWalk may not capture higher-order proximity, so it loses the global information. It also depends on the datasets.

## 5 Conclusion

We present a novel method for network embedding that preserves the local and global structure of the network. To capture the global structure and accelerate the efficiency of state-of-the-art methods, we introduce a neighborhood similarity-based graph compression method. We combine the vertices with common neighbors into super-node. Then we apply network representation learning on the compressed graph so that we can reduce the run time and also capture the global structure. As a first method, we project the embedding of super-nodes to original nodes without refinement. In the second part, we relearn the representation of the network with assigning the super-nodes embedding to its’ original vertices as initial features and update this using the baseline method. In this way, we combine the local structure with the global structure of the network. While the first method provides efficiency with learning on the small compressed graph, the second method provides effectiveness with incorporating global information into embedding with the compressed graph. NECL and NECL-RF are a general meta-strategies that can be used to improve the efficiency and effectiveness of many state-of-the-art graph embedding method. We use three popular state-of-the-art network embedding methods DeepWalk, Node2vec, and LINE as a baseline. Experimental results on various real-world graph show the effectiveness and efficiency of our methods on challenging multi-label and multi-class classification tasks for all these three baseline methods.

The future work of our NECL and NECL-RF could be using different refinement methods of graph embedding. We can apply different neural network models without relearning the whole network to refine the embedding which we get from the compressed graph. Another extension could be done by using different clustering methods or similarity measurements to compressed the graph and use other baseline methods.

## Data Availability Statement

The original contributions presented in the study are included in the article/Supplementary Material, further inquiries can be directed to the corresponding authors.

## Author Contributions

Conceived and designed the experiments: MI and EA Performed the experiments: MI , GJ, and EA. Analyzed the data: MI, GJ, EA, FT, and MA. Wrote the paper: MI, FT, GJ, EA, and MA.

## Funding

This work was partially done by the author Ginger Johnson while she attended the Big Data Analytics REU program at Oklahoma State University supported by the National Science Foundation under Grant No. 1659645.

## Conflict of Interest

The authors declare that the research was conducted in the absence of any commercial or financial relationships that could be construed as a potential conflict of interest.
